# miR-154-5p Functions as an Important Regulator of Angiotensin II-Mediated Heart Remodeling

**DOI:** 10.1155/2019/8768164

**Published:** 2019-09-12

**Authors:** Que Wang, Xiaoxue Yu, Lin Dou, Xiuqing Huang, Kaiyi Zhu, Jun Guo, Mingjing Yan, Siming Wang, Yong Man, Weiqing Tang, Tao Shen, Jian Li

**Affiliations:** ^1^Peking University Fifth School of Clinical Medicine, Beijing 100730, China; ^2^The MOH Key Laboratory of Geriatrics, Beijing Hospital, National Center of Gerontology, Beijing 100730, China; ^3^Shanxi Medical University, Taiyuan, Shanxi 030001, China

## Abstract

Chronic hypertension, valvular heart disease, and heart infarction cause cardiac remodeling and potentially lead to a series of pathological and structural changes in the left ventricular myocardium and a progressive decrease in heart function. Angiotensin II (AngII) plays a key role in the onset and development of cardiac remodeling. Many microRNAs (miRNAs), including miR-154-5p, may be involved in the development of cardiac remolding, but the underlying molecular mechanisms remain unclear. We aimed to characterize the function of miR-154-5p and reveal its mechanisms in cardiac remodeling induced by AngII. First, angiotensin II led to concurrent increases in miR-154-5p expression and cardiac remodeling in adult C57BL/6J mice. Second, overexpression of miR-154-5p to a level similar to that induced by AngII was sufficient to trigger cardiomyocyte hypertrophy and apoptosis, which is associated with profound activation of oxidative stress and inflammation. Treatment with a miR-154-5p inhibitor noticeably reversed these changes. Third, miR-154-5p directly inhibited arylsulfatase B (Arsb) expression by interacting with its 3′-UTR and promoted cardiomyocyte hypertrophy and apoptosis. Lastly, the angiotensin type 1 receptor blocker telmisartan attenuated AngII-induced cardiac hypertrophy, apoptosis, and fibrosis by blocking the increase in miR-154-5p expression. Moreover, upon miR-154-5p overexpression in isolated cardiomyocytes, the protective effect of telmisartan was partially abolished. Based on these results, increased cardiac miR-154-5p expression is both necessary and sufficient for AngII-induced cardiomyocyte hypertrophy and apoptosis, suggesting that the upregulation of miR-154-5p may be a crucial pathological factor and a potential therapeutic target for cardiac remodeling.

## 1. Introduction

Cardiac remodeling is an adaptive response to pathophysiological stimuli, such as ischemia/reperfusion or excessive mechanical load, and includes multiple molecular and cellular processes. Initially, cardiac remodeling may serve as a compensatory response; however, it slowly progresses to a decompensatory effect on heart function [1]. The mechanisms of pathological cardiac remodeling mainly include cardiomyocyte hypertrophy in response to both mechanical and neurohumoral triggers, cardiomyocyte loss mediated by cell death pathways, and fibrosis leading to the accumulation of an excess extracellular matrix [2]. Angiotensin II (AngII), a core component of the renin-angiotensin system (RAS), plays a key role in the onset and development of cardiac remodeling. Two receptors for AngII are expressed in the heart: AT1 and AT2. AT1 receptors have been proposed to mediate most of the pathophysiological effects of AngII, whereas the functions of AT2 receptors remain controversial. Therefore, many antihypertensive drugs have been designed to block the AT1 receptor [3].

MicroRNAs (miRNAs), a group of conserved, endogenous, and noncoding RNAs (19–25 nucleotides in length), negatively regulate the expression of their target genes by directly binding to the 3′-UTR of their target mRNAs. By inhibiting the translation or degradation of mRNAs, miRNAs regulate the expression of their target genes posttranscriptionally [4]. Moreover, miRNAs are involved in a wide range of biological processes. To date, miRNAs have been reported to exhibit abnormal expression and regulate organ function in the cardiovascular system in subjects with some pathological conditions [5]. For instance, muscle-specific miR-133 regulates protein levels by inhibiting the translation of target genes involved in cardiac contractility and hypertrophy [6]. By targeting most extracellular matrix-related mRNAs, both miR-29 and miR-30 are strongly related to fibrosis [7].

miR-154-5p is a conserved miRNA in many species. According to previous studies, miR-154-5p is related to cell proliferation and metastasis in glioblastoma, renal cell carcinoma, and non-small-cell lung cancer [8–10]. Notably, miR-154-5p is related to the activation of the Wnt signaling pathway [11, 12]. In the cardiovascular system, the overexpression of miR-154-5p increases the activation of cardiac fibroblasts in a pressure overload model [11, 13]. However, little is known about the role of miR-154-5p in cardiac disorders induced by the neurohumoral trigger AngII.

The Arsb (arylsulfatase B) gene is located on chromosome 13 in mice and is expressed at higher levels in the heart. Arsb is an enzyme in lysosome and cell membranes that is required for the degradation of sulfated glycosaminoglycans (4-sulfate groups from chondroitin-4-sulfate and dermatan sulfate) at the nonreducing end of the sulfated glycosaminoglycan (GAG) chain [14, 15]. Mucopolysaccharidosis VI (MPS VI; Maroteaux-Lamy syndrome) is a genetic lysosomal disorder caused by Arsb deficiency, and sulfated GAGs are accumulated throughout the body, leading to pathological processes [16]. However, the effects of Arsb on the heart that have been reported to date only include some alterations in a normal physiological process in subjects with MPS VI. GAGs are accumulated in endothelial cells, cardiomyocytes, and fibroblasts, influencing the structure and function of the endocardium, myocardium, valves, and coronary arteries [15]. Cardiomyopathy occurs in 20% to 30% of patients with MPS VI [17] who present with left atrial enlargement or left ventricular (LV) hypertrophy, and most patients die of heart failure in a few decades [18]. However, the mechanism by which the Arsb deficiency induces cardiomyopathy remains unclear.

Based on these facts, we hypothesized that miR-154-5p might be involved in the mechanism regulating the pathophysiological process of AngII-induced cardiac remodeling and might be a potential therapeutic target for cardiovascular disease.

## 2. Materials and Methods

### 2.1. Mouse Model of Angiotensin II-Induced Heart Hypertrophy

All animal experiments conformed to the protocols approved by the Beijing Hospital, Ministry of Health Animal Use and Care Committee, and to the *Guide for the Care and Use of Laboratory Animals* (NIH publication #85-23, revised in 1996).

10 to 12-week-old male C57BL/6J mice were anesthetized with 1–1.5% isoflurane in oxygen. AngII (1, 1.5, 2.5, or 4 mg/kg per day) in saline or saline alone were delivered by infusion through an osmotic minipump (ALZET, Cupertino, CA; DURECT, Cupertino, CA) for 14 days after the operation to subcutaneously insert the pump. All mice were identically housed and fed the same chow.

1, 3, 7, or 14 days after AngII infusion (2.5 mg/kg per day), mice were anesthetized with 3-4% isoflurane and euthanized by cervical dislocation. The hearts were removed and weighed, and the ratio of the heart to the total body weight was determined. RNA and proteins were extracted, and the hearts were sectioned for further studies.

### 2.2. Oral Administration of Drugs

Telmisartan (Boehringer Ingelheim Pharmaceuticals, Germany) was purchased commercially. Male C57BL/6J mice were randomly divided into the following four groups: sham+saline, sham+telmisartan, AngII+saline, and AngII+ telmisartan. Telmisartan was dissolved in saline, and equal volumes of a freshly prepared telmisartan solution or saline (0.2 ml) were administered to mice once daily through an oral gavage starting 3 days before the operation to the end of the experiments. The dosages of telmisartan were 21.4 mg kg^−1^day^−1^. Drug dosages were selected based on the Bios formula, clinically relevant concentrations, and a preliminary study [19].

### 2.3. Culture and Treatment of Mouse Cardiomyocytes

The procedures for culturing primary cardiomyocytes were essentially the same as those described in a previous study [20]. Neonatal mouse ventricular myocytes (NMVMs) were isolated from 1-3-day-old C57BL/6J mice by digestion with the combination of trypsin and collagenase type II. Cardiomyocytes were plated at a density of 6.6 × 10^4^ cells/cm^2^ in Dulbecco's Modified Eagle's Medium (DMEM; Sigma, USA) supplemented with 10% fetal bovine serum (FBS; HyClone, USA) in the presence of 0.1 mM 5-bromo-2-deoxyuridine and incubated at 37°C in humidified air with 5% CO_2_. The sequences of negative control, miR-154-5p mimics, microRNA inhibitor negative control, and 154-5p inhibitor were as follows (5′–3′): negative control sense, UUCUCCGAACGUGUCACGUTT; negative control antisense, ACGUGACACGUUCGGAGAATT; miR-154-5p mimics, UAGGUUAUCCGUUGCCUUCGAAGGCAACACGGAUAACCUAUU; microRNA inhibitor negative control, CAGUACUUUUGUGUAGUACAA; and miR-154-5p inhibitor, CGAAGGCAACACGGAUAACCUA. miRNA oligos were purchased from Sangon Biotech (Shanghai). Arsb plasmid and vector were obtained from OriGene Technologies (Beijing). Scramble and si-Arsb sequence were synthesized from Santa Cruz Biotechnology. Gene transfer mediated by an adenovirus or Lipofectamine RNAiMAX (Invitrogen, USA) was performed after 24 h of quiescent culture in DMEM supplemented with 1% FBS, and then cells were treated with 100 nM AngII for another 24 h. Pretreatments were performed by adding telmisartan (a selective AT1 receptor antagonist) before exposing cells to AngII.

### 2.4. Luciferase Assay

The 3′-untranslated region (3′-UTR) of target genes and their mutant variant were synthesized and digested with SacI and XhoI to generate reporter vectors containing miRNA-binding sites (Shengong Co., China). The fragment was inserted into the pmir-GLO luciferase reporter vector (Promega, USA) using previously described methods [21]. For the luciferase assay, miR-154-5p mimics were cotransfected with the luciferase reporters containing Arsb-3′-UTR, mutant-Arsb-3′UTR, or pmir-GLO luciferase reporter vector into HEK-293A cells using a transfection reagent (Vigofect, Vigorous Biotechnology, China). Forty-eight hours after transfection, luciferase activity was detected using the Dual-Luciferase Reporter Assay System (Promega), according to the manufacturer's protocol.

### 2.5. RNA Extraction and Real-Time PCR

Total RNA was extracted from cells and tissues using TRIzol (Invitrogen, Carlsbad, CA, USA) and reverse-transcribed into first-strand cDNAs using a kit from New England Biolabs. Real-time PCR was performed using the iCycler IQ system (Bio-Rad) and a reaction mixture containing SYBR Green (TaKaRa, Japan) according to the manufacturer's protocol. The PCR program was essentially the same as described in a previous study [22]. The data are presented as the averages of 4–6 independent experiments.

### 2.6. Western Blotting

Western blotting was used to assess the levels of the Arsb protein with a primary antibody against Arsb (Proteintech, USA). In a subset of the experiments, phospho-p38 MAPK, p38 MAPK, phospho-JNK, JNK, phospho-STAT3, STAT3, GAPDH, and caspase-3 (Cell Signaling Technology, USA) levels were measured with specific antibodies. ImageJ software (NIH) was used for the densitometry analysis.

### 2.7. Histology, TUNEL Staining, and Immunofluorescence Staining

Histology and immunofluorescence staining were performed on adult hearts and cardiomyocytes. For the histological analysis, 7 *μ*m sections or fixed cells were incubated with the primary antibodies overnight at 4°C. After washes with 0.25% Triton X-100 in PBS, sections or cells were incubated with secondary antibodies for 2 h. TUNEL staining was performed in the heart cryosections or fixed cells using a cell death detection kit from Roche, and the nuclei were then stained with 10 mM Hoechst33342 as previously described [22]. The location of NF-*κ*B was determined by staining samples with a rabbit anti-NF-*κ*B (p65) antibody and an anti-rabbit secondary antibody (Cell Signaling Technology, USA).

### 2.8. WGA and Masson's Trichrome Staining

Cross-sectional areas of heart cryosection were measured using wheat germ agglutinin (Alexa Fluor™ 488 Conjugate) staining (Thermo Fisher Scientific, USA), and heart fibrosis was analyzed with the Masson's Trichrome Staining Kit from Solarbio according to the manufacturer's protocol. All sections were imaged using a microscope.

### 2.9. Actin-Tracker Staining

Cells were washed with PBS, fixed with 4% paraformaldehyde for 10 min, and then permeabilized with 0.1% Triton X-100 for 10 min. Cells were incubated with Actin-Tracker Green (Beyotime, Shanghai, China) at room temperature for 1 hour. Then, the nuclei of cells were stained with Hoechst33342 and cells were subsequently visualized under a fluorescence microscope (Olympus BX51, USA). Adobe Photoshop CS6 (Adobe Systems, USA) was used to prepare merged images.

### 2.10. In Situ Detection of Reactive Oxygen Species (ROS)

Frozen, unfixed, and living cardiomyocytes were stained with 10 *μ*mol/L dihydroethidium (DHE, fluorescent probe for detecting the intracellular superoxide anion level, Sigma) in a dark, humidified chamber at 37°C for 30 min to evaluate ROS production in cardiomyocytes in situ. ROS generation was indicated by red fluorescence detected with a fluorescence microscope and quantified using the ImageJ software (NIH).

### 2.11. Data Analysis and Statistics

Values are presented as the means ± SEM of at least three experiments. Data were analyzed using the statistical software GraphPad Prism 6.0 (GraphPad Software, CA, USA). The differences between two groups were analyzed using Student's *t*-test, and multiple comparisons were analyzed using one-way ANOVA with Bonferroni's correction. Differences between groups were considered significant at *P* < 0.05.

## 3. Results

### 3.1. Angiotensin II Led to Concurrent Increases in miR-154-5p Expression and Cardiac Remodeling In Vivo and In Vitro

**Figure 1:**
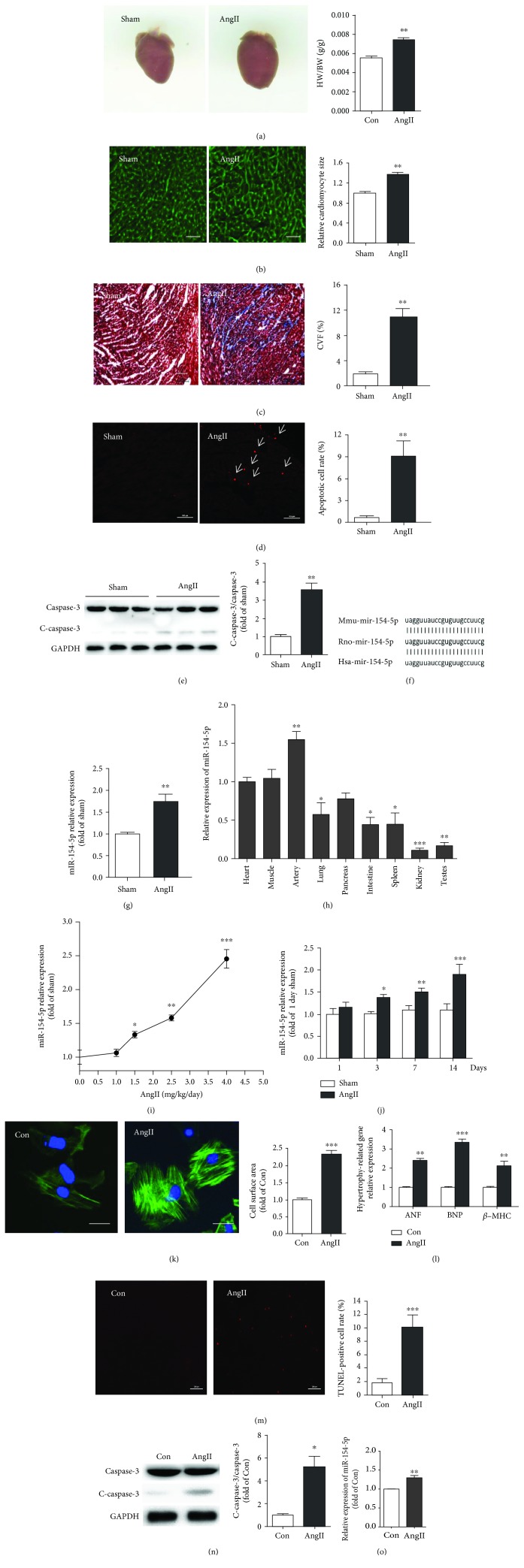
miR-154-5p is upregulated during AngII-induced cardiac remodeling. (a) Representative images of mouse hearts and the ratio of heart weight to body weight (HW/BW) in sham- and AngII-treated (2.5 mg/kg per day, 14 days) mice (*n* = 6). In this time point, the heart was in the period of cardiac hypertrophy. (b) Histological analysis of the surface area of mouse cardiomyocytes using WGA staining (*n* = 5). (c) Images of Masson's trichrome-stained sections of the mouse heart (*n* = 5). (d) Images of TUNEL staining and the average data obtained from heart sections (*n* = 5). (e) Cleaved caspase-3 and caspase-3 expression, as assayed by Western blotting (*n* = 6). (f) Conservation of the miR-154-5p sequence between mice, rats, and humans is shown. (g) The cardiac expression of miR-154-5p in AngII-treated and sham-treated mice. (h) Distribution of miR-154-5p in various tissues from C57BL/6J mice (*n* = 6). miR-154-5p expression is upregulated in a dose-dependent (i) and time-dependent (j) manner in AngII-treated mice (*n* = 5). (k) The surface area of cardiomyocytes (control group and treated 100 nM AngII for 24 h) was identified using *α*-actinin staining (green), and nuclei were stained with Hoechst33342 (blue) (*n* = 50). (l) Expression of the ANF, BNP, and *β*-MHC mRNAs in cardiomyocytes (*n* = 3). (m) Images of TUNEL staining in cardiomyocytes and quantitative analysis (*n* = 4). (n) Levels of cleaved caspase-3 and caspase-3 in AngII-treated and control cardiomyocytes (*n* = 3). (o) The expression of miR-154-5p in AngII-treated and control cardiomyocytes (*n* = 3). Data are presented as the means ± SEM. ^∗^*P* < 0.05, ^∗∗^*P* < 0.01, and ^∗∗∗^*P* < 0.001.

Male C57BL/6J mice were implanted with osmotic mini pumps that delivered AngII (2.5 mg/kg per day) for 14 days to induce cardiac remodeling and saline as the negative control. The representative images were generated after AngII treatment for 14 days. In this time point, the heart was in the period of cardiac hypertrophy. Echocardiography showed that there was no significant difference in cardiac function between the AngII- and sham-treated mice (data not shown). Higher heart/body weight ratios were observed in the AngII-treated mice than in the saline-treated mice (Figure 1(a)). Histological staining revealed a significant increase in the cardiomyocyte area and fibrosis of ventricular tissues in the AngII-treated hearts (Figures 1(b) and 1(c)). Moreover, the apoptosis rate was also significantly increased in AngII-treated mice compared with control mice, as examined by TUNEL staining and Western blotting for caspase-3 (Figures 1(d) and 1(e)). Meanwhile, to understand which miRNA is involved in the process of cardiac remodeling, we used a miRNA microarray approach to identify changes in miRNA expression and determine which miRNA was involved in the process of AngII-induced cardiac remodeling. The expression of 268 miRNAs was increased (>2-fold) in the AngII-treated mice compared to the saline-treated mice (data not shown). We examined the expression and functions of the miRNA candidates in AngII-treated mice. Among the miRNAs identified in this screen, miR-154-5p expression was confirmed to be significantly increased in the AngII-treated mouse model, suggesting that it might be involved in the development of cardiac remodeling (Figure 1(g)).

Next, upon analyzing the sequence, miR-154-5p has been highly conserved during evolution (Figure 1(f)). We examined the distribution of miR-154-5p expression in mouse tissues. Real-time PCR analyses of multiple adult mouse tissues revealed that miR-154-5p is widely expressed in many organs and tissues and is expressed at high levels in the heart (Figure 1(h)). Then, we examined miR-154-5p expression in adult male mice treated with different doses of AngII at different time points. AngII dose- and time-dependently increased miR-154-5p expression in adult mouse models (Figures 1(i)and 1(j)).

Moreover, we established a cardiomyocyte hypertrophy model by treating NMVMs with 100 nM AngII for 24 h. Compared to the control group, AngII-treated cardiomyocytes showed marked increases in the cell size and the expression of the ANF, BNP, and *β*-MHC mRNAs (Figures 1(k) and 1(l)). In addition, AngII also induced cardiomyocyte apoptosis, as shown by TUNEL staining and Western blotting for caspase-3 (Figures 1(m) and 1(n)). Similar to its upregulation in AngII-induced mouse hearts, miR-154-5p expression was also upregulated in AngII-treated cardiomyocytes (Figure 1(o)).

### 3.2. miR-154-5p Was Sufficient to Trigger Cardiomyocyte Hypertrophy and Apoptosis, Which Were Associated with a Profound Increase in Oxidative Stress and Inflammation

**Figure 2:**
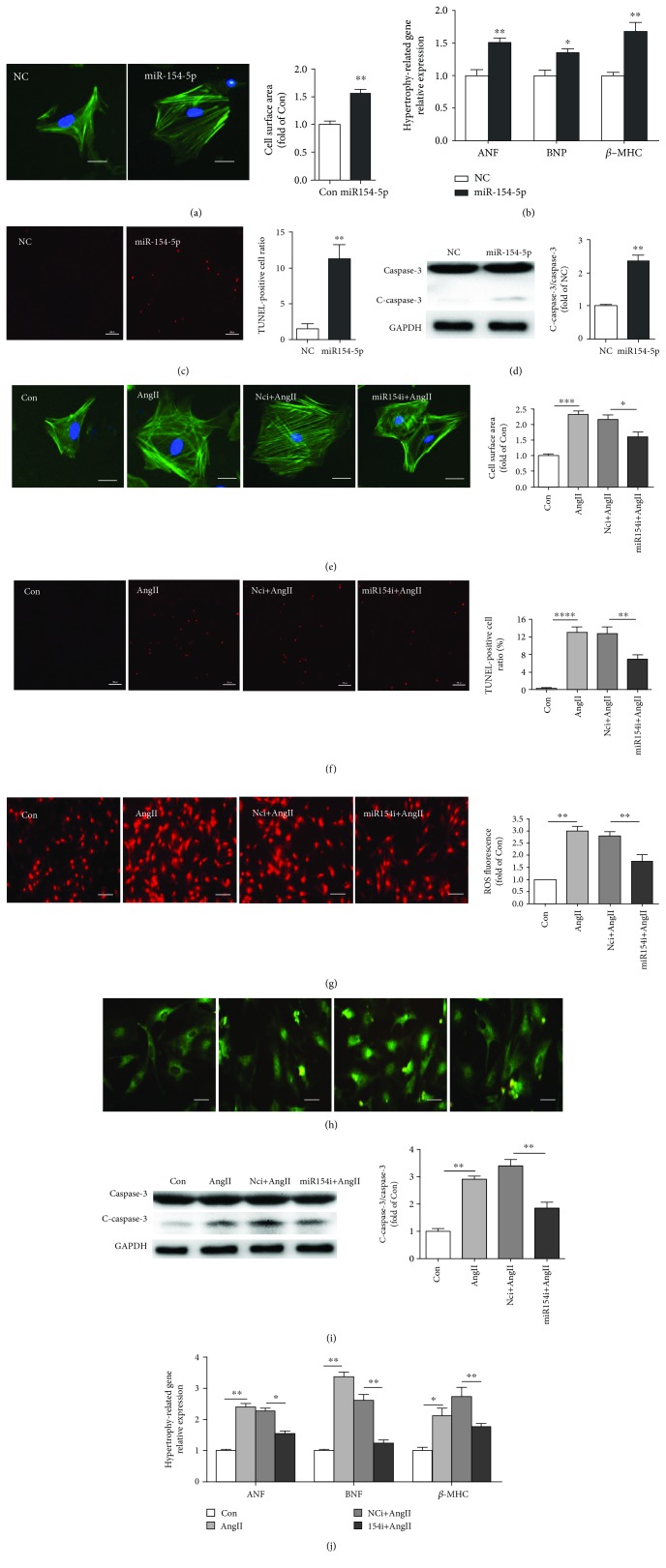
miR-154-5p induces cardiac hypertrophy and apoptosis by promoting oxidative stress and inflammatory reactions in cardiomyocytes. (a) Images of the cell area labeled with Actin-Tracker Green (*n* = 50). (b) Results from the real-time PCR analysis of the expression of the ANF, BNP, and *β*-MHC mRNAs in cardiomyocytes transfected with normal control scrambled RNA (NC) and miR-154-5p mimics (*n* = 3). (c) Images of TUNEL staining and quantification of the percentage of apoptotic cells (*n* = 4). (d) Western blots and the quantification of cleaved caspase-3 and caspase-3 levels (*n* = 3). (e) Images of the cell area labeled with Actin-Tracker Green staining and quantitative analysis of the control, AngII, normal control inhibitor RNA with AngII (NCi+AngII), and miR-154 inhibitor with AngII (miR-154i+AngII) groups (*n* = 50). (f) Images and quantitative analysis of TUNEL staining (*n* = 4). (g) DHE staining was used to analyze ROS levels (*n* = 4). (h) Images of double immunofluorescence staining showing the NF-*κ*B (green) localization. (i) Western blot and quantitative analysis of caspase-3 and cleaved caspase-3 levels (*n* = 3). (j) Average levels of ANF, BNP, and *β*-MHC mRNA expression (*n* = 3). ^∗^*P* < 0.05, ^∗∗^*P* < 0.01, ^∗∗∗^*P* < 0.001, and ^∗∗∗∗^*P* < 0.0001.

We overexpressed miR-154-5p using RNA mimics and knocked down its expression using an miR-154-5p inhibitor (an antisense sequence of nucleotide) in cardiomyocytes to investigate the function of miR-154-5p in cardiomyocytes. The overexpression of miR-154-5p alone significantly increased cardiomyocyte hypertrophy, while the miR-154-5p inhibitor attenuated AngII-induced cardiomyocyte hypertrophy (Figures 2(a) and 2(e)). Moreover, real-time-PCR analysis revealed increased expression of hypertrophy-related genes, including ANF, BNP, and *β*-MHC, in cells transfected with miR-154-5p mimics, while the miR-154-5p inhibitor blunted the expression of these genes in NMVMs stimulated with AngII (Figures 2(b) and 2(j)).

Interestingly, miR-154-5p was also potentially involved in cardiomyocyte injury and apoptosis. Overexpression of miR-154-5p induced cardiomyocyte apoptosis, and knockdown of miR-154-5p attenuated AngII-induced apoptosis, as analyzed by TUNEL staining and Western blotting for caspase-3 (Figures 2(c), 2(d), 2(f), and 2(i)).

Furthermore, miR-154-5p also regulated ROS production and the NF-*κ*B signaling pathway in primary cardiomyocytes. The miR-154-5p inhibitor reversed AngII-induced ROS production, as shown by DHE staining and NF-*κ*B activation assayed by determining the translocation of the p65 protein (green fluorescence) from the cytoplasm to the nucleus (Figures 2(g) and 2(h)).

Based on these results, miR-154-5p was sufficient to trigger cardiomyocyte hypertrophy and apoptosis, which were associated with a substantial increase in oxidative stress and inflammation in vitro.

### 3.3. mir-154-5p Directly Targeted Arsb by Interacting with Its 3′-UTR

**Figure 3:**
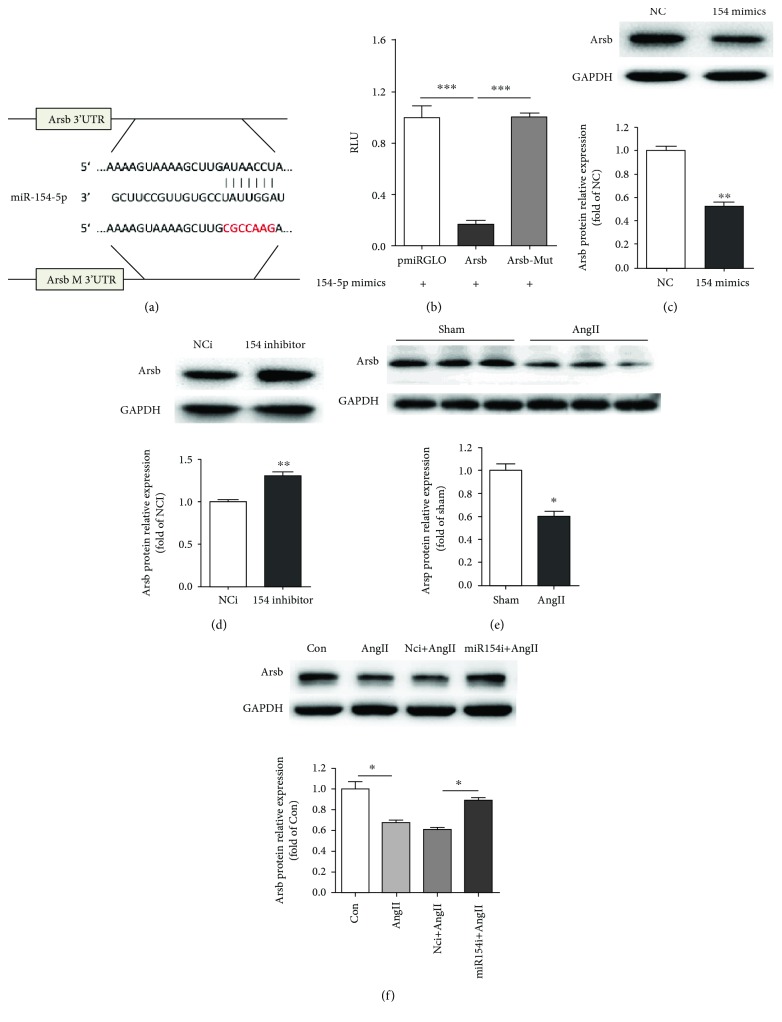
miR-154-5p directly targets Arsb. (a) A binding site for miR-154-5p in the 3′-UTR of Arsb was analyzed using miRNA target prediction databases, including miRBase, miRanda, TargetScan, and PicTar. The mutated sequence of the binging site is marked in red. (b) Luciferase activity was analyzed in HEK-293A cells 24 h after transfection with the indicated plasmids: miR-154-5p+vectors, miR-154-5p+Arsb 3′UTR, and miR-154-5p+mutated Arsb 3′UTR (*n* = 4). (c, d) The level of the Arsb protein was examined using Western blot analysis in the NC, miR-154-5p mimic, NCi, and miR154-5p inhibitor groups (*n* = 3). (e) Protein level of Arsb in sham and AngII mice explored by Western blot (*n* = 3). (f) Protein level of Arsb in AngII-treated primary cardiomyocytes transfected with NCi and miR-154-5p inhibitor (*n* = 3). ^∗^*P* < 0.05, ^∗∗^*P* < 0.01, and ^∗∗∗^*P* < 0.001.

We next sought to identify the putative miR-154-5p targets using the target prediction programs miRBase, miRanda, TargetScan, and PicTar. We screened 16 candidate genes with miRNA binding sites in their 3′-UTRs. We synthesized the 3′-UTR of each candidate gene and the mutated binding sites into a pmiRGLO vector to further verify whether miR-154-5p directly bound to these genes. The Arsb gene might be a direct downstream target of miR-154-5p in cardiomyocytes (Figure 3(a)). When cotransfected with miR-154-5p mimics into HEK293A cells, the relative luciferase activity of the Arsb 3′-UTR reporter was significantly suppressed compared with the control vector in the luciferase reporter assay. This phenotype was reversed when the miR-154-5p binding site in the 3′-UTR of Arsb was mutated (Figure 3(b)).

Western blots also showed a significant decrease in Arsb levels transfected with miR-154-5p mimics and increased Arsb expression transfected with the miR-154-5p inhibitor in cardiomyocytes (Figures 3(c) and 3(d)). Moreover, in AngII-induced mice and AngII-treated cardiomyocytes, the Arsb protein level was reduced and the miR-154-5p inhibitor reversed the Arsb level in primary cardiomyocytes (Figures 3(e) and 3(f)).

Thus, miR-154-5p inhibited Arsb expression by directly binding to its 3′-UTR.

### 3.4. miR-154-5p Promoted Cardiomyocyte Hypertrophy and Apoptosis by Inhibiting Arsb Expression and Activating the p38 MAPK/JNK/STAT3 Pathway

**Figure 4:**
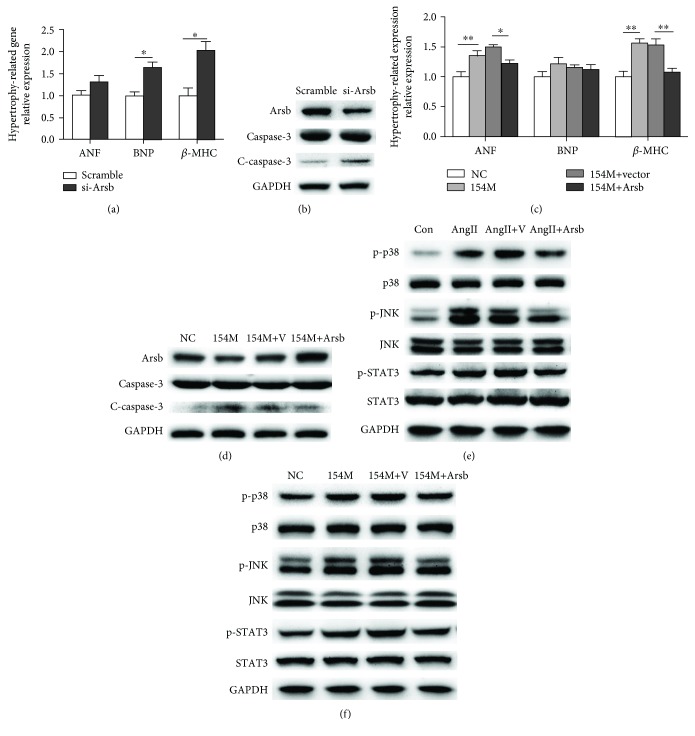
Upregulation of Arsb reverses the effects of miR-154-5p on cardiomyocyte hypertrophy and apoptosis. (a) Expression of markers of cardiac hypertrophy in cardiomyocytes transfected with scramble, si-Arsb, as measured using real-time PCR (*n* = 3). (b) Western blot images showing the levels of Arsb, cleaved caspase-3, and caspase-3 in cardiomyocytes subjected to different treatments (*n* = 3). (c) Expression of markers of cardiac hypertrophy in cardiomyocytes transfected with NC, miR-154-5p, miR-154-5p+vector, and miR-154-5p+Arsb, as measured using real-time PCR (*n* = 3). (d) Western blot images showing the levels of Arsb, cleaved caspase-3, and caspase-3 in cardiomyocytes subjected to different treatments (*n* = 3). (e, f) Western blot images showing the levels of the p-JNK, JNK, p-p38, p38, p-STAT3, and STAT3 proteins in the Con, AngII, AngII+vector, and AngII+Arsb groups (*n* = 3) and in the NC, miR-154-p, miR-154-5p+vector, and miR-154-5p+Arsb groups (*n* = 3).

We transfected NMVMs with miR-154-5p mimics and Arsb to investigate the role of the miR-154-5p/Arsb axis in cardiac hypertrophy and apoptosis. Firstly, we determined the role of Arsb in cardiomyocyte hypertrophy and apoptosis. As shown in Figures 4(a) and 4(b), Arsb knockdown could induce increased cardiac hypertrophy markers and cardiomyocyte apoptosis. Furthermore, miR-154-5p induced hypertrophy and apoptosis, while Arsb overexpression abolished these effects, as indicated by real-time PCR and Western blots (Figures 4(c) and 4(d)). Based on these data, Arsb overexpression attenuated cardiac hypertrophy and apoptosis induced by miR-154-5p. (Western blot densitometric analyses in Supplemental [Supplementary-material supplementary-material-1]).

Next, AngII induced cardiac remodeling and increased the levels of phospho-p38, phospho-JNK, and phospho-STAT3, which are important members of pathways regulating cardiac hypertrophy and apoptosis, while Arsb overexpression inhibited the increase in NMVMs (Figure 4(e)). Moreover, Arsb overexpression also reversed the effect of miR-154-5p on the activation of the p38 MAPK/JNK/STAT3 pathway in primary cultured cardiomyocytes (Figure 4(f)). Therefore, Arsb was one of the key regulators of cardiomyocyte hypertrophy and apoptosis induced by miR-154-5p (Western blot densitometric analyses in Supplemental [Supplementary-material supplementary-material-1]).

Based on these findings, miR-154-5p promoted cardiac hypertrophy and apoptosis mainly by inhibiting Arsb expression, subsequently attenuating the activation of the p38 MAPK/JNK/STAT3 pathway.

### 3.5. Telmisartan Attenuated AngII-Induced Cardiac Remodeling and miR-154-5p Upregulation In Vivo and In Vitro

**Figure 5:**
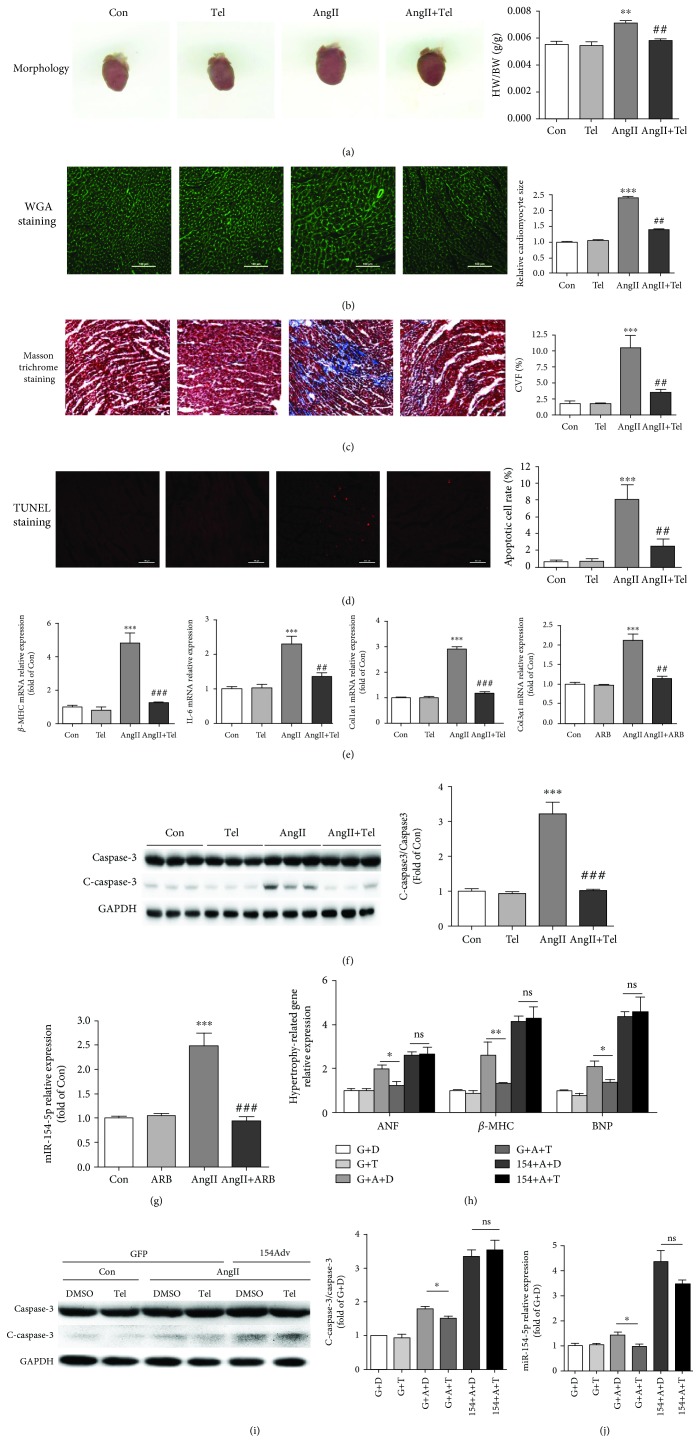
miR-154-5p is involved in AngII-induced cardiac remodeling mediated by the AT1 receptor in vivo and in vitro. (a) Representative images of mouse hearts and the ratio of heart weight to body weight (HW/BW) in control (Con), telmisartan- (Tel-), AngII-, and AngII+telmisartan- (AngII+Tel-) treated mice (*n* = 6). (b) Histological analysis of the surface area of cardiomyocytes in the Con, Tel-, AngII-, and AngII+Tel-treated groups using WGA staining (*n* = 8). (c) Images of Masson's trichrome-stained sections of the hearts from Con, Tel-, AngII-, and AngII+Tel-treated mice (*n* = 5). (d) TUNEL staining and quantitative analysis of sections of the hearts from Con, Tel-, AngII-, and AngII+Tel-treated mice (*n* = 6). (e) Expression of markers of cardiac remodeling in the myocardium of different groups of treated mice, as measured using real-time PCR (*n* = 5). (f) Western blot and quantitative analysis of cleaved caspase-3 and caspase-3 levels in the hearts of Con, Tel-, AngII-, and AngII+Tel-treated mice (*n* = 5). (g) Cardiac expression of miR-154-5p, as detected using real-time PCR (*n* = 3). ^∗^*P* < 0.05 compared with the Con group; ^#^*P* < 0.05 compared with the AngII group. (h) Expression of markers of cardiac hypertrophy in cardiomyocytes treated with GFP+DMSO (G+D), GFP+telmisartan (G+T), GFP+AngII+DMSO (G+A+D), GFP+AngII+telmisartan (G+A+T), miR-154-5p+AngII+DMSO (154+A+D), or miR-154-5p+AngII+telmisartan (154+A+T) (*n* = 3). (i) Western blots showing the levels of cleaved caspase-3 and caspase-3 (*n* = 4). (j) Expression of miR-154-5p in cells subjected to different treatments, as measured using real-time PCR (*n* = 3). ^∗^*P* < 0.05, ^∗∗^*P* < 0.01, ^∗∗∗^*P* < 0.001, and ns = not significant.

Although AngII is a well-documented inducer of cardiac hypertrophy, apoptosis, and fibrosis, which are generally mediated by the AT1 receptor but not AT2, its underlying molecular mechanisms, including a possible role for miRNAs, remain to be completely elucidated. We used telmisartan, an angiotensin type 1 receptor blocker (ARB), to block the function of AngII in WT mice and determine how AngII regulates miR-154-5p to induce cardiac hypertrophy and apoptosis. A pretreatment with telmisartan significantly decreased the AngII-induced increases in the HW/BW, cardiomyocyte area (Figures 5(a) and 5(b)), and expression of hypertrophy-, inflammation-, and fibrosis-related genes (*β*-MHC, Il-6, Col1*α*1, and Col3*α*1) in WT mice (Figure 5(e)). Moreover, the pretreatment with telmisartan efficiently attenuated AngII-induced cardiomyocyte apoptosis and cardiac fibrosis (Figures 5(c), 5(d), and 5(f)). The AngII-induced increase in miR-154-5p expression in the heart was almost completely blocked by telmisartan (Figure 5(g)).

We also confirmed these findings in vitro using cultured NMVMs treated with telmisartan and AngII (100 nM). As shown in Figure 5(j), the AngII-induced increase in miR-154-5p expression in cardiomyocytes was completely blocked by telmisartan. Similar to the telmisartan-induced inhibition of Ang-II-induced upregulation of genes in hearts, the expression of hypertrophy-related genes and levels of apoptosis were decreased in telmisartan+AngII-treated cardiomyocytes compared with the AngII-treated group. In particular, upon miR-154-5p overexpression, the protective effect of telmisartan on AngII-induced cardiac hypertrophy and apoptosis was partially abolished (Figures 5(h) and 5(i)).

Thus, miR-154-5p was one of the major regulators of AngII-induced cardiac remodeling through the AT1 receptor.

## 4. Discussion

Here, miR-154-5p played a key role in cardiac hypertrophy and cell apoptosis. First, AngII dose- and time-dependently increased miR-154-5p expression in AngII-treated male C57BL/6J mice. Upregulation of miR-154-5p directly increased oxidative stress, inflammation, pathological hypertrophy, and cardiomyocyte apoptosis in vitro. Additionally, the inhibition of miR-154-5p significantly reduced these responses in AngII-treated cardiomyocytes. Furthermore, we confirmed that Arsb is involved in the responses induced by miR-154-5p as a target and participates in modulating the p38 MAPK/JNK/STAT3 pathway. Arsb was validated as a target using bioinformatics analyses and molecular biological methods. The reintroduction of Arsb attenuated miR-154-5p-induced cardiac hypertrophy and cardiomyocyte apoptosis. Moreover, the AT1 receptor blocker telmisartan completely inhibited the AngII-mediated increase in miR-154-5p expression in vivo and in vitro. Thus, miR154-5p is one of the key regulators of AngII-mediated cardiac remodeling and may be a therapeutic target in the treatment of cardiovascular diseases.

Previous studies of miR-154-5p have mainly focused on cancer research. Our finding that miR-154-5p induces apoptosis is consistent with the results reported by Lin et al., Chen and Gao, and Pang et al., who showed that miR-154-5p increased apoptosis in skin squamous cell carcinoma, non-small-cell lung cancer, and hepatocellular carcinoma [10, 23, 24]. Moreover, the role of miR-154-5p in tumors is still controversial. miR-154-5p inhibits tumor growth and metastasis in glioma and prostate cancer, but it promotes proliferation in renal cell carcinoma [8, 9]. In addition, miR-154-5p increases the proliferation of cardiac fibroblasts and lung fibroblasts and alters the cell cycle progression (S phase to G1 phase) of DU145 and/or PC3 cells [11, 13, 25]. Cardiomyocytes are nonproliferating cells, but the cells described above are all proliferating cells. Cell proliferation is a very important cellular process involving multiple factors. These factors may play other roles in nonproliferating cells through different pathways. Therefore, in nonproliferating cells such as cardiomyocytes, rather than increasing proliferation, miR-154-5p may promote hypertrophy to adapt to the changes in the microenvironment. However, this hypothesis requires further testing in the future.

Bernardo et al. reported the role of miR-154 in a different mouse model of pressure overload [13]. There are actually many differences between our study and the results reported. The causes of myocardial remodeling include mechanical factors and neurohormone factors. Kent and McDermott and Malhotra et al. believe that mechanical factors and AngII-induced myocardial remodeling are activated by different signaling pathways in regulating cardiomyocytes [26, 27]. Similarly, studies have shown that the effects of these two factors are mutually reinforcing. Mechanical stimulation can induce cardiomyocytes to increase the secretion of AngII by paracrine and act on adjacent cardiomyocytes to promote hypertrophy [28]. The AT1 receptor is the major effector receptor for AngII in cardiomyocytes, and the AT1 receptor inhibitor has been widely used for cardiac diseases in clinical practice. In recent years, studies have also shown that mechanical factors can also directly act on the AT1 receptor, promoting cardiomyocyte hypertrophy, but the mechanism remains unclear [29]. Therefore, we believe that our study is aimed at defining the role of miR-154-5p in the AngII-induced cardiac hypertrophy model, which clearly demonstrates the regulatory mechanism of miR-154-5p in cardiac hypertrophy. There are differences between our study and that published by Bernardo et al. (1) In our study, we not only demonstrated the role of miR-154-5p at the animal level but also excluded the influence of other cells in the cardiac tissue, demonstrating its regulation in cardiomyocytes. (2) During remodeling, cardiomyocytes mainly manifested as an increase in cardiomyocyte hypertrophy and death. We introduced the analysis of apoptosis, confirming the regulation of miR-154-5p on the cell death process. (3) We investigated the role of miR-154-5p by determining the target gene Arsb. (4) We first proposed and confirmed the regulation of Arsb in cardiac hypertrophy. (5) By using an AT1 receptor inhibitor, it was confirmed that miR-154-5p is a new drug therapeutic target.

Our finding that Arsb is a target of miR-154-5p and participates in the development of cardiomyocyte hypertrophy is important, consistent with the cardiac pathological phenomena in patients with MPS VI. We first identified the significant role of decreased Arsb expression in cardiomyocyte hypertrophy based on strong molecular, cellular, and animal evidence. Reduced Arsb expression is involved in colonic and prostatic malignancies, causing increased inflammation [30]. In human prostate stem and epithelial cells, Arsb silencing increases the expression of C4S and EGFR (Her1/ErbB1). Then, the increased expression of EGFR increases c-Jun N-terminal kinase (JNK) activity [31]. Likewise, Arsb regulates the expression of GPNMB (transmembrane glycoprotein NMB) in HepG2 cells by increasing the levels of phospho-p38 [32]. In our study, Arsb regulated cardiac hypertrophy via the p38 MAPK/JNK pathway, consistent with previous studies. In addition, Arsb participated in the mechanism regulating cardiac hypertrophy and apoptosis by modulating the STAT3 pathway, which may be a new pathway activated by an Arsb deficiency or a new therapeutic target for MPS VI in cardiomyocytes.

AT1 receptor blockers (ARBs) are highly effective drugs that are available to treat hypertension in the clinic. They bind to the AT1 receptor and inhibit AngII-induced receptor activation [33]. Currently, many clinical trials and meta-analysis have suggested that the ARBs exert strong protective effects on the cardiovascular system. ARBs are some of the most effective drugs at reducing cardiac hypertrophy and protecting heart function in patients with essential hypertension. The therapeutic effects of ARBs are better than the *β*-blocker atenolol and a calcium channel blocker. ARBs confer greater beneficial effects on hypertensive patients with cardiac hypertrophy [34–36]. AT1 participates in the activation of multiple signaling pathways, including the ERK, p38, JNK, and STAT3 pathways, and ARBs effectively block the pathways [37–39]. However, the specific intracellular components mediating signal transduction remain unclear. In the present study, miR-154-5p and Arsb were involved in the protective effect of the ARB telmisartan on cardiomyocytes. This discovery provides additional evidence that improves our understanding of the pharmacological mechanism of ARBs and a potential therapeutic strategy for cardiac remodeling treatment.

## 5. Conclusion

In summary, the present study is the first to show a marked increase in miR-154-5p expression induced by AngII-mediated cardiac remodeling. The upregulation of miR-154-5p is both necessary and sufficient to trigger oxidative stress, inflammation, cardiac hypertrophy, and apoptosis. These findings have defined an important role for a noncoding RNA in cardiac remodeling in response to AngII-induced heart injury. We conclude that the AngII/miR-154-5p/Arsb axis is a crucial cascade for the development of cardiac remodeling, and it regulates the oxidative stress and the inflammatory response. Because cardiomyocyte hypertrophy and apoptosis are key cellular processes involved in the development of heart failure, the present findings may implicate novel drug targets and therapeutic strategies for hitherto lethal heart diseases, including myocardial infarction, congestive heart hypertrophy, and heart failure.

## Data Availability

All data generated or analyzed during this study are included in this published article.

## Supplementary Materials

Supplementary Materials Supplemental Figure 1: Western blot densitometric analyses. (A) Western blot densitometric analyses showing the levels of Arsb, cleaved caspase-3, and caspase-3 in cardiomyocytes transfected with scramble and si-Arsb sequences (*n* = 3). (B) Western blot densitometric analyses showing the levels of Arsb, cleaved caspase-3, and caspase-3 in cardiomyocytes transfected with NC, miR-154-5p, miR-154-5p+vector, and miR-154-5p+Arsb (*n* = 3). (C, D) Western blot densitometric analyses showing the levels of the p-JNK, JNK, p-p38, p38, p-STAT3, and STAT3 proteins in the Con, AngII, AngII+vector, and AngII+Arsb groups (*n* = 3) and in the NC, miR-154-p, miR-154-5p+vector, miR-154-5p+Arsb groups (*n* = 3). ^∗^*P* < 0.05, ^∗∗^*P* < 0.01, and ^∗∗∗^*P* < 0.001. Supplemental Table 1: 3′UTR sequences of 16 target genes. The synthetic sequences are shown in the table (binding sites and mutated sequences are underlined).

## References

[1] Orenes-Piñero E., Montoro-García S., Patel J. V., Valdés M., Marín F., Lip G. Y. H. (2013). Role of microRNAs in cardiac remodelling: new insights and future perspectives. *International Journal of Cardiology*.

[2] Burchfield J. S., Xie M., Hill J. A. (2013). Pathological ventricular remodeling: mechanisms: part 1 of 2. *Circulation*.

[3] Zhu Y. C., Zhu Y. Z., Lu N., Wang M. J., Wang Y. X., Yao T. (2003). Role of angiotensin AT_1_ and AT_2_ receptors in cardiac hypertrophy and cardiac remodelling. *Clinical and Experimental Pharmacology and Physiology*.

[4] Romaine S. P. R., Tomaszewski M., Condorelli G., Samani N. J. (2015). MicroRNAs in cardiovascular disease: an introduction for clinicians. *Heart*.

[5] Thum T., Condorelli G. (2015). Long noncoding RNAs and microRNAs in cardiovascular pathophysiology. *Circulation Research*.

[6] Carè A., Catalucci D., Felicetti F. (2007). *MicroRNA-133* controls cardiac hypertrophy. *Nature Medicine*.

[7] van Rooij E., Sutherland L. B., Thatcher J. E. (2008). Dysregulation of microRNAs after myocardial infarction reveals a role of miR-29 in cardiac fibrosis. *Proceedings of the National Academy of Sciences of the United States of America*.

[8] Wang X., Sun S., Tong X. (2017). MiRNA-154-5p inhibits cell proliferation and metastasis by targeting PIWIL1 in glioblastoma. *Brain Research*.

[9] Lin C., Li Z., Chen P. (2018). Oncogene miR-154-5p regulates cellular function and acts as a molecular marker with poor prognosis in renal cell carcinoma. *Life Sciences*.

[10] Lin X., Yang Z., Zhang P., Shao G. (2015). miR-154 suppresses non-small cell lung cancer growth in vitro and in vivo. *Oncology Reports*.

[11] Sun L. Y., Bie Z. D., Zhang C. H., Li H., Li L. D., Yang J. (2016). miR-154 directly suppresses *DKK2* to activate Wnt signaling pathway and enhance activation of cardiac fibroblasts. *Cell Biology International*.

[12] Zhou H., Zhang M., Yuan H., Zheng W., Meng C., Zhao D. (2016). MicroRNA-154 functions as a tumor suppressor in osteosarcoma by targeting Wnt5a. *Oncology Reports*.

[13] Bernardo B. C., Nguyen S. S., Gao X. M. (2016). Inhibition of miR-154 protects against cardiac dysfunction and fibrosis in a mouse model of pressure overload. *Scientific Reports*.

[14] Bartolomeo R., Polishchuk E. V., Volpi N., Polishchuk R. S., Auricchio A. (2013). Pharmacological read-through of nonsense ARSB mutations as a potential therapeutic approach for mucopolysaccharidosis VI. *Journal of Inherited Metabolic Disease*.

[15] Golda A., Jurecka A., Tylki-Szymanska A. (2012). Cardiovascular manifestations of mucopolysaccharidosis type VI (Maroteaux–Lamy syndrome). *International Journal of Cardiology*.

[16] Bhattacharyya S., Feferman L., Tobacman J. K. (2014). Arylsulfatase B regulates versican expression by galectin-3 and AP-1 mediated transcriptional effects. *Oncogene*.

[17] Chen M. R., Lin S. P., Hwang H. K., Yu C. H. (2005). Cardiovascular changes in mucopolysaccharidoses in Taiwan. *Acta Cardiologica*.

[18] Strauch O. F., Stypmann J., Reinheckel T., Martinez E., Haverkamp W., Peters C. (2003). Cardiac and ocular pathologies in a mouse model of mucopolysaccharidosis type VI. *Pediatric Research*.

[19] Wang X., Ye Y., Gong H. (2016). The effects of different angiotensin II type 1 receptor blockers on the regulation of the ACE-AngII-AT1 and ACE2-Ang(1–7)-Mas axes in pressure overload-induced cardiac remodeling in male mice. *Journal of Molecular and Cellular Cardiology*.

[20] Shen T., Zheng M., Cao C. (2007). Mitofusin-2 is a major determinant of oxidative stress-mediated heart muscle cell apoptosis. *Journal of Biological Chemistry*.

[21] Dou L., Wang S., Sui X. (2015). miR-301a mediates the effect of IL-6 on the AKT/GSK pathway and hepatic glycogenesis by regulating PTEN expression. *Cellular Physiology and Biochemistry*.

[22] Shen T., Yang C., Ding L. (2013). Tbx20 functions as an important regulator of estrogen-mediated cardiomyocyte protection during oxidative stress. *International Journal of Cardiology*.

[23] Chen H. Q., Gao D. (2018). Inhibitory effect of microRNA-154 targeting WHSC1 on cell proliferation of human skin squamous cell carcinoma through mediating the P53 signaling pathway. *The International Journal of Biochemistry & Cell Biology*.

[24] Pang X., Huang K., Zhang Q., Zhang Y., Niu J. (2015). miR-154 targeting ZEB2 in hepatocellular carcinoma functions as a potential tumor suppressor. *Oncology Reports*.

[25] Milosevic J., Pandit K., Magister M. (2012). Profibrotic role of miR-154 in pulmonary fibrosis. *American Journal of Respiratory Cell and Molecular Biology*.

[26] Kent R. L., McDermott P. J. (1996). Passive load and angiotensin II evoke differential responses of gene expression and protein synthesis in cardiac myocytes. *Circulation Research*.

[27] Malhotra R., Sadoshima J., Brosius F. C., Izumo S. (1999). Mechanical stretch and angiotensin II differentially upregulate the renin-angiotensin system in cardiac myocytes in vitro. *Circulation Research*.

[28] Harada K., Komuro I., Shiojima I. (1998). Pressure overload induces cardiac hypertrophy in angiotensin II type 1A receptor knockout mice. *Circulation*.

[29] Sadoshima J., Xu Y., Slayter H. S., Izumo S. (1993). Autocrine release of angiotensin II mediates stretch-induced hypertrophy of cardiac myocytes in vitro. *Cell*.

[30] Bhattacharyya S., Feferman L., Tobacman J. K. (2014). Increased expression of colonic Wnt9A through Sp1-mediated transcriptional effects involving arylsulfatase B, chondroitin 4-sulfate, and galectin-3. *Journal of Biological Chemistry*.

[31] Bhattacharyya S., Feferman L., Han X. (2018). Decline in arylsulfatase B expression increases EGFR expression by inhibiting the protein-tyrosine phosphatase SHP2 and activating JNK in prostate cells. *Journal of Biological Chemistry*.

[32] Bhattacharyya S., Feferman L., Tobacman J. K. (2016). Inhibition of phosphatase activity follows decline in sulfatase activity and leads to transcriptional effects through sustained phosphorylation of transcription factor MITF. *PLoS One*.

[33] Zaman M. A., Oparil S., Calhoun D. A. (2002). Drugs targeting the renin–angiotensin–aldosterone system. *Nature Reviews Drug Discovery*.

[34] Klingbeil A. U., Schneider M., Martus P., Messerli F. H., Schmieder R. E. (2003). A meta-analysis of the effects of treatment on left ventricular mass in essential hypertension. *The American Journal of Medicine*.

[35] Kjeldsen S. E., Dahlöf B., Devereux R. B. (2002). Effects of losartan on cardiovascular morbidity and mortality in patients with isolated systolic hypertension and left ventricular hypertrophy: a Losartan Intervention for Endpoint Reduction (LIFE) substudy. *JAMA*.

[36] Ogihara T., Fujimoto A., Nakao K., Saruta T. (2008). ARB candesartan and CCB amlodipine in hypertensive patients: the CASE-J trial. *Expert Review of Cardiovascular Therapy*.

[37] Yamazaki T., Komuro I., Kudoh S. (1995). Angiotensin II partly mediates mechanical stress–induced cardiac hypertrophy. *Circulation Research*.

[38] Nishida M., Tanabe S., Maruyama Y. (2005). G*α*12/13- and reactive oxygen species-dependent activation of c-Jun NH_2_-terminal kinase and p38 mitogen-activated protein kinase by angiotensin receptor stimulation in rat neonatal cardiomyocytes. *Journal of Biological Chemistry*.

[39] Bueno O. F., Molkentin J. D. (2002). Involvement of extracellular signal-regulated kinases 1/2 in cardiac hypertrophy and cell death. *Circulation Research*.

