# Supplementary material for: miR-154-5p Functions as an Important Regulator of Angiotensin II-Mediated Heart Remodeling
Source: Oxid Med Cell Longev. 2019 Sep 12;2019:8768164. doi: 10.1155/2019/8768164 (PMC6757276; doi:10.1155/2019/8768164)
Supplement: Supplementary Materials — Supplemental Figure 1: Western blot densitometric analyses. (A) Western blot densitometric analyses showing the levels of Arsb, cleaved caspase-3, and caspase-3 in cardiomyocytes transfected with scramble and si-Arsb sequences (n = 3). (B) Western blot densitometric analyses showing the levels of Arsb, cleaved caspase-3, and caspase-3 in cardiomyocytes transfected with NC, miR-154-5p, miR-154-5p+vector, and miR-154-5p+Arsb (n = 3). (C, D) Western blot densitometric analyses showing the levels of the p-JNK, JNK, p-p38, p38, p-STAT3, and STAT3 proteins in the Con, AngII, AngII+vector, and AngII+Arsb groups (n = 3) and in the NC, miR-154-p, miR-154-5p+vector, miR-154-5p+Arsb groups (n = 3). ∗P < 0.05, ∗∗P < 0.01, and ∗∗∗P < 0.001. Supplemental Table 1: 3′UTR sequences of 16 target genes. The synthetic sequences are shown in the table (binding sites and mutated sequences are underlined). [file 8768164.f1.pdf]

Supplemental figure. 1

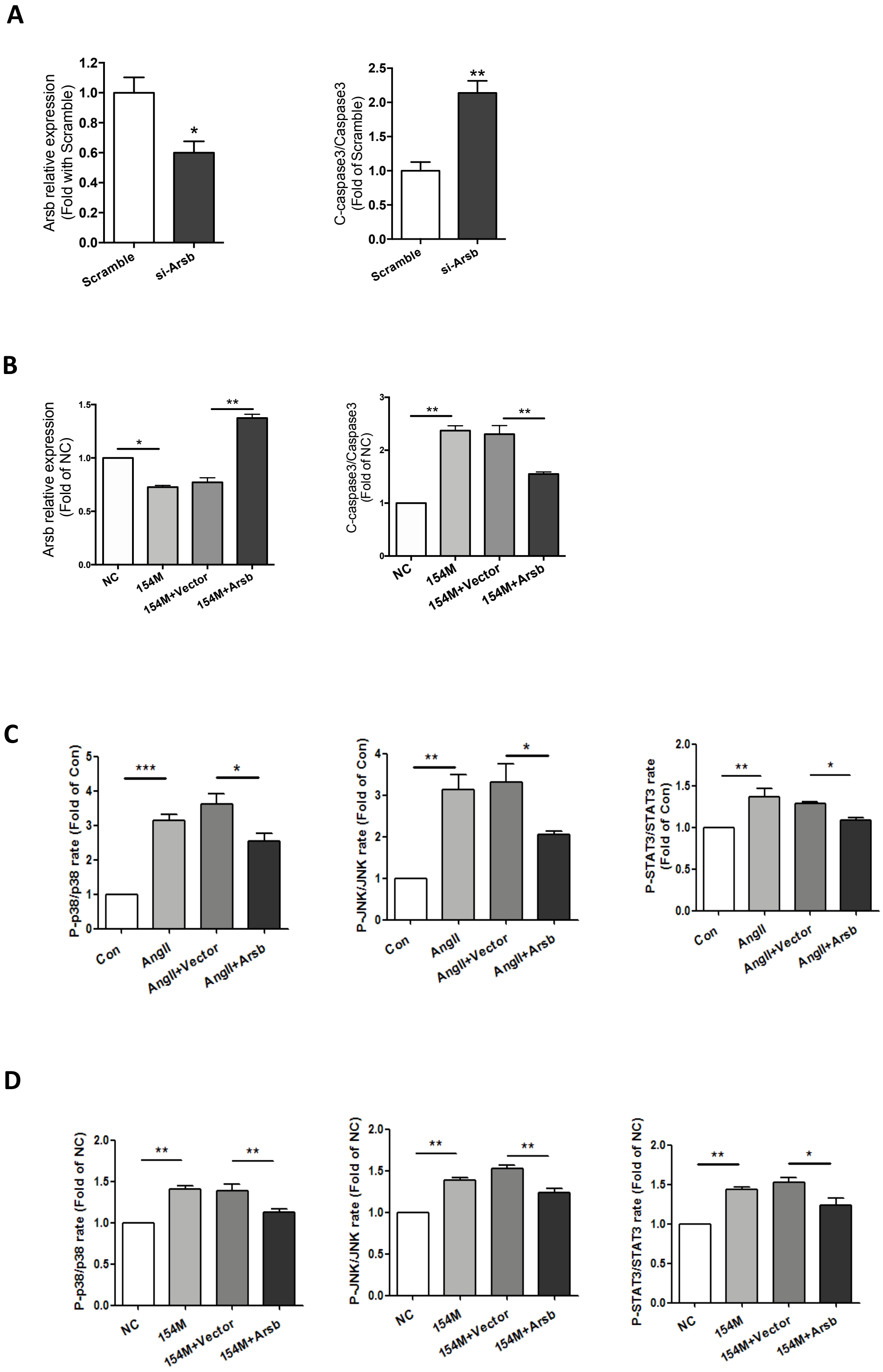

Supplemental Table.1

Candidate gene name	Original sequence	Mutated sequences
Arsb	...AAAAGUAAAAGCUUGAUAACCUA...	...AAAAGUAAAAGCUUGCGCCAAGA...
Kcna2	...UUUAAAACACAGGAGAUAACCUA...	...UUUAAAACACAGGAGCGCCAAGA...
IL-6	...UAUAGUUUUGAAAUGAUAACCUA...	...UAUAGUUUUGAAAUGCGCCAAGA...
Dnm1l	...CUAAAUGAUCCAAUGAUAACCUA...	...CUAAAUGAUCCAAUGCGCCAAGA...
Camk4	...AGAUGGUUAUAAAAGAUAACCUG...	...AGAUGGUUAUAAAAGCGCCAAGG...
Plekha1	...CCUUGUCAUCAGUAGUAACCUAU...	...CCUUGUCAUCAGUAGGCCAAGAU...
Apln	...CCCCCAAGCCCCAUAAUAACCUG...	...CCCCCAAGCCCCAUACGCCAAGG...
Rps6ka5	...ACAGUUGUGUUAAUGAUAACCUC...	...ACAGUUGUGUUAAUGCGCCAAGC...
Cops8	...AUUUUAUACACGCUUAUAACCUG...	...AUUUUAUACACGCUUCGCCAAGG...
Psmf1	...AAUAAAUAGCACUUUAUAACCUG...	...AAUAAAUAGCACUUUCGCCAAGG...
Id2	...UGUAUAGUGGCAGAAAUAACCUA...	...UGUAUAGUGGCAGAACGCCAAGA...
Mmp20	...ACAGAUCCAGGAAAAAUAACCUA...	...ACAGAUCCAGGAAAACGCCAAGA...
Nphs2	...AGACAUGCCCACAGGAUAACCUA...	...AGACAUGCCCACAGGCGCCAAGA...
Dpp8	...AAUAAUUUUUCAAGGAUAACCUA...	...AAUAAUUUUUCAAGGCGCCAAGA...
Prr11	...GUUAAUUGAGAUGGGAUAACCUA...	...GUUAAUUGAGAUGGGCGCCAAGA...
Bach1	...CCUCGUGGGCCGUGGAUAACCUA...	...CCUCGUGGGCCGUGGCGCCAAGA...
